# Neuroimaging Detectable Differences between Parkinson's Disease Motor Subtypes: A Systematic Review

**DOI:** 10.1002/mdc3.13107

**Published:** 2020-11-06

**Authors:** Jackson Tyler Boonstra, Stijn Michielse, Yasin Temel, Govert Hoogland, Ali Jahanshahi

**Affiliations:** ^1^ Department of Neurosurgery, School for Mental Health and Neuroscience (MHeNS) Maastricht University Medical Center Maastricht The Netherlands

**Keywords:** Parkinson's disease (PD), neuroanatomy, neuroimaging, motor subtypes, tremor‐dominant (TD)

## Abstract

**Background:**

The neuroanatomical substrates of Parkinson's disease (PD) with tremor‐dominance (TD) and those with non‐tremor dominance (nTD), postural instability and gait difficulty (PIGD), and akinetic‐rigid (AR) are not fully differentiated. A better understanding of symptom specific pathoanatomical markers of PD subtypes may result in earlier diagnosis and more tailored treatment. Here, we aim to give an overview of the neuroimaging literature that compared PD motor subtypes.

**Methods:**

A systematic literature review on neuroimaging studies of PD subtypes was conducted according to the Preferred Reporting Items for Systematic Reviews and Meta‐Analyses (PRISMA) guidelines. Search terms submitted to the PubMed database included: “Parkinson's disease”, “MRI” and “motor subtypes” (TD, nTD, PIGD, AR). The results are first discussed from macro to micro level of organization (i.e., (1) structural; (2) functional; and (3) molecular) and then by applied imaging methodology.

**Findings:**

Several neuroimaging methods including diffusion imaging and positron emission tomography (PET) distinguish specific PD motor subtypes well, although findings are mixed. Furthermore, our review demonstrates that nTD‐PD patients have more severe neuroalterations compared to TD‐PD patients. More specifically, nTD‐PD patients have deficits within striato‐thalamo‐cortical (STC) circuitry and other thalamocortical projections related to cognitive and sensorimotor function, while TD‐PD patients tend to have greater cerebello‐thalamo‐cortical (CTC) circuitry dysfunction.

**Conclusions:**

Based on the literature, STC and CTC circuitry deficits seem to be the key features of PD and the subtypes. Future research should make greater use of multimodal neuroimaging and techniques that have higher sensitivity in delineating subcortical structures involved in motor diseases.

Parkinson's disease (PD) is a progressive and complex neurodegenerative disorder. Patients with PD show highly heterogeneous clinical characteristics developing tremors and/or kinesia paradoxa, an umbrella term for non‐tremor motor symptoms including absence of movement (akinesia), decreased amplitude of movement (hypokinesia), and slowness in movement execution (bradykinesia).^1^

PD motor subtypes, including tremor‐dominant (TD) and non‐tremor dominant (nTD) (often characterized by postural instability & gait difficulty (PIGD) and akinetic‐rigid (AR)), suggest different pathophysiologies.^2^, ^3^, ^4^, ^5^ These motor subtypes are mainly determined by the Movement Disorder Society Unified Parkinson's Disease Rating Scale (MDS‐UPDRS), a comprehensive 50‐question assessment of both motor and non‐motor symptoms associated with PD.^6^ Similarly, the original UPDRS is also utilized in PD research and can be used for the same purpose. The ratio of the mean tremor scores (eight items from Part III) to the mean of the PIGD scores (five items from Part III) is used to delineate TD patients (ratio ≥ 1.5), from PIGD patients (ratio ≤ 1), and from intermediate or ‘mixed‐type’ patients (ratios >1.0 and < 1.5).^7^ Additionally, subscores of TD and AR can be derived by averaging symptom specific questions from Part III.^8^ These classifications help to clinically distinguish PD subtypes and allow for steady investigations of symptom specific alterations.

On a pathological level, PD is characterized by progressive degeneration of intertwined subcortical dopaminergic nigrostriatal systems,^9^, ^10^ Lewy body aggregations, and depletion of dopamine in the striatum^11^, ^12^, ^13^ all of which can be identified via postmortem histology. Compared to TD, AR patients have shown more severe cell loss in the substantia nigra (SN) and such cell loss was shown to negatively correlate with AR symptom severity.^14^ nTD patients have shown more severe cell loss in the ventrolateral part of the substantia nigra pars compacta (SNc) that projects to the dorsal putamen, causing inhibition of the glutamatergic thalamo‐cortical (*direct*) pathway and reduced cortical activation, while in contrast, TD patients show more severe neuronal loss in the medial, rather than in the lateral SNc that projects to the lateral putamen, caudate nucleus, ventromedial thalamus, and rubral areas (*indirect pathway*) leading to hyperactivity of thalamo‐motor projections.^14^ In this light, nTD is thought to be due more to abnormal basal ganglia (BG) output while TD evolves additional downstream compensatory mechanisms.^5^ Previous studies using diverse neuroimaging methodologies have been utilized to understand PD circuitopathies. However, the full extent of the neuroanatomical and neurofunctional differences between the PD motor subtypes TD and nTD that can be seen with neuroimaging are poorly understood.^10^, ^15^, ^16^, ^17^ Further differentiating motor‐subtypes of PD through neuroimaging will increase the ability to monitor progression and identify at risk populations, possibly even at an asymptomatic phase of PD,^18^, ^19^, ^20^ and work to improve localization and targeting for non‐invasive and invasive neuromodulation therapies.^21^, ^22^ Here, previous research that used neuroimaging techniques to characterize structural and functional variances between TD and nTD subtypes of PD are consolidated. First, an overview of imaging studies related to the neuroanatomical, functional, and neurochemical basis of TD and nTD PD is given. These are followed by descriptions of limitations that occur within each imaging methodology when applied to PD. Lastly, potential hypotheses are addressed that may be tested by neuroimaging PD motor subtypes, their clinical implications, and how this may increase insight into neurobiological underpinnings.

## Methods

### Literature Selection

This review was conducted according to the Preferred Reporting Items for Systematic Reviews and Meta‐Analyses (PRISMA) guidelines. We evaluated human neuroimaging studies on PD subtypes TD, nTD, PIGD, and AR published in international English written peer reviewed journals up to May 2020. A PubMed search based on various dictions of Parkinson's disease (PD) (MeSH), neuroimaging technique (MRI) (MeSH), and PD subtypes (TD (MeSH), nTD, PIGD, AR) were applied (see Supplementary Material File S1). This resulted in 546 publications that were independently reviewed by two assessors. Full text of the articles were reviewed and additional articles were found via reference sections. Seventy‐five articles were included in the final analysis.

### Inclusion Criteria

1. Study analyzed human data and was published in English.
2. Study reported the proportion of PD patients with TD‐PD and nTD‐PD.
3. Comparative neuroimaging analysis had been carried out directly concerning TD‐PD versus nTD‐PD.

### Data Extraction

Relevant data obtained and collected using a data extraction spreadsheet and grouped per neuroimaging modality included:

- Primary author and year of publication
- Imaging method
- MRI strength and vendor
- Number of participants in PD subtypes
- Key findings

## Results

### Structural Imaging

#### *Structural Imaging Techniques*

MR‐based techniques allow for visualization of the microstructural anatomy of brain tissue. One processing method on high‐resolution 3DT1 sequences is called voxel based morphometry (VBM) that can compare local concentrations of gray matter between groups of subjects.^23^ Other techniques include diffusion‐weighted imaging (DWI), a method to measure the diffusion of water molecules within the brain, and diffusion tensor imaging (DTI), a paradigm that analyses the three‐dimensional shape of such diffusion and allows for visualization of fiber tracts. Additional quantitative structural MR‐based techniques include neuromelanin sensitive MRI (NM‐MRI) used to detect a product of dopamine metabolism called neuromelanin, age‐related white matter changes (ARWMC) which are hyperintense lesions observed on T2‐weighted MR images, and leukoaraiosis or white matter hyperintensities (WMH), an abnormal change in white matter near lateral ventricles.^24^

### Cortical and Subcortical Volumes

#### *Gray Matter*

Measurements of gray matter (GM) can potentially reveal more about neural functions that underpin particular symptomologies (Table 1). In reference 25, researchers found that TD had less GM atrophy in frontal, parietal, occipital, and temporal lobes, as well as in the caudate nucleus and the cerebellar culmen, and later found TD had larger GM volumes (GMV) in the amygdala and globus pallidus (GP) compared to PIGD but with no cerebellar differences.^26^ In 2017, one group found lower GMV in the frontal cortex of TD compared to PIGD, but this difference did not hold true when nuisance covariates of disease severity, disease duration, and medication were controlled for.^27^ Another study showed TD to have significantly larger GMV along the lateral border of the right thalamus compared to nTD.^28^ The GM degeneration in frontal regions could be the underlying cause or consequence of the greater cognitive decline that is commonly seen within PIGD^29^ while cognitive decline itself may also feed into gait difficulties, as fall risk was previously found to be related to the motor‐cognitive interdependence of executive function.^30^ The amygdala GMV changes could also underlie numerous affective (non‐motor) symptoms in nTD, including depression, apathy, and anxiety.^31^ Similarly, as loss of smell is a prodromal sign of PD,^1^ TD having larger olfactory bulb volumes compared to nTD^32^ could point towards differing symptoms between subtypes.

**TABLE 1:** *Differences with structural MRI between PD motor subtypes*

Study	MRI Strength	PD	TD	nTD	PIGD	AR	Mixed	HC	Findings
**Gray matter**									
(Al‐Bachari et al., 2017^27^	3 T Philips		21		24		6		TD trended to have lower GMV in the frontal cortex compared to PIGD. TD had lower WML volume compared to PIGD.
(Altinayar et al., 2014)^32^	1.5 T Siemens		27	14				19	TD had larger olfactory bulb volumes than nTD.
(Benninger et al., 2009)^28^	3 T Philips		14	10					TD had lower GMV in the mainly right posterior part of the cerebellar quadrangular lobe. TD had larger GMV along the lateral border of the right thalamus compared to nTD.
(Piccinin et al., 2017)^33^	3 T Philips		44			19			TD had decreased GMV in the left lobule VIIIa compared to AR.
(Rosenberg‐Katz et al., 2013)^25^	3 T GE		29		30				TD had higher GMV in frontal, parietal, occipital, temporal lobes, right caudate nucleus, amygdala, and the bilateral cerebellar culmen and left declive compared to PIGD.
(Rosenberg‐Katz et al., 2016)^26^	3 T GE		30		30			28	TD had larger GMV in amygdala and globus pallidus compared to PIGD.
**Cortical Thickness**									
(Danti et al., 2015)^40^	1.5 T Siemens		22		14			18	MCI‐TD showed no significant cortical thinning compared to MCI‐PIGD.
(Herb et al., 2016)^38^	3 T Philips		45		74	58			TD had greater cortical thickness than PIGD in the dorsolateral frontal lobes, anterior temporal lobes, and cuneus, and precuneus. PIGD had reduced cortical thickness compared to AR in multiple areas. No cortical distinctions were shown between TD and AR.
(Karunanayaka et al., 2016)^39^	3 T Siemens		15			17		24	No differences in cortical thickness were seen between AR, TD, and controls in the DMN.
(Linder et al., 2009)^43^	1.5 T Philips		23		35		8	30	TD showed no difference in MRI parameters compared to PIGD.
(Nyberg et al., 2015)^41^	3 T GE		12		9			20	TD had no significant subcortical volume differences compared to PIGD.
(Prodoehl et al., 2013)^44^	3 T GE		10	10				20	TD did not differ in cortical or subcortical GMV or WMV compared to nTD.
(Tessa et al., 2008)^45^	1.5 T Siemens		13			11	3	16	TD did not differ in total brain GMV or WMV compared to AR or healthy controls.
(Vervoort et al., 2016)^69^	3 T Philips		16		39			19	No volumetric differences in caudate, putamen and pallidum were shown between TD and PIGD. No shape alterations in right caudate and the bilateral putamen and pallidum were seen between TD and PIGD.
**White matter**									
(Bohnen et al., 2011)^48^	3 T Philips		25		36		10		TD had lower WMH compared to PIGD.
(Fang et al., 2020)^56^	3 T Siemens	21			23			20	PIGD patients showed significantly higher white matter lesion load in the caudate and lateral and third ventricle when compared to non‐PIGD.
(Herman et al., 2013)^54^	3 T GE		42		62		4		TD did not differ in mean number of voxels with WMHs compared to PIGD.
(Lee et al., 2009)^55^	1.5 T GE		75		54				TD were independently associated with lower leukoaraiosis grade compared to PIGD.
(Moccia et al., 2016)^49^	1.5 T Philips		27, 35, 36		18, 20, 22		18, 8, 4		TD had lower ARWMC compared to PIGD at baseline and at 2 and 4 years follow up.
(Shen et al., 2019)^52^	1.5 T Philips		40		136				TD/non‐PIGD patients had lower white matter hyperintensity scores compared to PIGD.
(Wan et al., 2019)^50^	3 T GE	38			89				PIGD showed more severe deep WMH in the frontal and occipital lobes when compared to non‐PIGD.
**Iron‐sensitive**									
(He et a., 2017)^2^	3 T GE		19			24		48	TD had significantly higher DN susceptibility values than AR.
(Jin et al., 2012)^59^	3 T GE		25			48	14	50	TD and AR had correlations between serum ceruloplasmin levels and nigral bilateral average phase values. No differences in the bilateral average phase values between TD, AR, and Mixed in the bilateral substantia nigra, globus pallidus, putamen, the head of caudate, and the red nucleus.
(Schneider et al., 2016)^60^	3 T Siemens	21			19			20	PIGD had lower SWI intensity values in putamen and globus pallidus compared to nPIGD.
(Xiang et al., 2017)^61^	3 T Philips		9		14			20	PIGD had more severe signal attenuation in the medial part of the substantia nigra pars compacta compared to TD.
**Other structural metrics**									
(Chiu et al., 2018)^92^	1.5 T, 3 T GE		48		22	64			PIGD was associated with a higher prevalence of cerebral microbleeds when compared to TD and AR.

Note: Rosenberg‐Katz et al., 2013 & 2016 report upon the same study sample. In Moccia et al., 2016, multiple numbers reflect baseline cohort and follow up cohorts at 2 and 4 years, respectively.

AR, akinetic‐rigid; ARWMC, age‐related white matter changes; DMN, default mode network; DN, dentate nucleus; GE, General Electric; GMV, gray matter volumes; HC, healthy controls; MCI, mild cognitive impairment; Mixed, mixed‐type/intermediate/indeterminate subtype; nPIGD, non postural instability and gait difficulty; nTD, non tremor‐dominant; PD, Parkinson's disease; PIGD, postural instability and gait difficulty; SWI, susceptibility weighted imaging; T, Tesla; TD, tremor‐dominant; WMH, white matter hyperintensity; WML, white matter lesions; WMV, white matter volumes.

Interestingly, TD had lower GMV in the posterior part of the right quadrangular lobe and in the declive of the cerebellum^28^ while a separate study showed TD had decreased GM in the cerebellular left lobule VIIIa compared to AR.^33^ Cerebellar atrophy could explain deficits within cerebello‐thalamo‐cortical (CTC) circuitry known to be deficient in TD patients^34^ as the cerebellum has shown to perceive tremor as a voluntary motor behavior and modulate tremor amplitude.^35^ The smaller pallidal, putamen, and caudate volumes in nTD are in line with the model of degenerating neurons in the cortico‐basal ganglia‐thalamo‐cortical loop that seem to be related to hypokinesia.^36^ Furthermore, increased thalamic and GP volumes found in TD suggest this regional enlargement indicates that TD are initially protected from a damaged basal ganglia‐thalamocortical circuitry and could potentially explain why the TD subtype does not experience PIGD symptoms associated with BG degeneration.^26^ These results are further supported by a recent lesion study showing PIGD patients have higher novel deep gray nuclear lesion load in the caudate compared to non‐PIGD and healthy controls (HC).^56^ GM analysis appears to support current PD circuitry models that underlie motor subtype differentiation of neuronal loss in key relay nuclei and stands as a valuable tool in the diagnostics and evaluation of PD subtypes.

#### *Cortical Thickness*

An important neuroanatomical aspect of PD is the thickness of gray matter in the cortex, as cortical thinning has shown to be primarily responsible for the reduction of cortical GMV.^37^ One study found reduced cortical thickness in PIGD patients compared to AR patients in areas including the bilateral frontal lobes, superior parietal cortices, and posterior cortical regions.^38^ The study reported PIGD to have reduced cortical thickness compared to TD in areas including the dorsolateral frontal lobes, anterior temporal lobes, and cuneus/precuneus, although no distinctions were seen when TD were compared to AR individuals or between PIGD and AR as the most pronounced cortical differences were between TD and PIGD patients localized to the left frontal region.

In contrast, a study using a smaller sample found that cortical thickness was similar between AR, TD, and healthy controls in specific brain regions part of the default mode network (DMN) such as the posterior cingulate cortex (PCC), the precuneus, the bilateral IPC, the medial prefrontal cortex (PFC), the anterior cingulate cortex (ACC), and the medial/lateral temporal lobe,^39^ while another study of PD patients with mild cognitive impairment (MCI) also showed similar cortical thinning amongst MCI‐TD compared to MCI‐PIGD.^40^ Another study showed that TD mean subcortical volumes were larger than PIGD in the putamen, caudate nucleus, GP, amygdala, and nucleus accumbens (NAc), and although these differences did not reach statistical significance, shape analysis resulting from local outward surface deviations revealed a significant difference in the right NAc shape between the two PD subtypes, mainly driven by the TD subtype, and the magnitude of the shape deviation was significantly correlated with MDS‐UPDRS TD and PIGD ratios suggesting that this NAc metric may hold as a neuroimaging biomarker for PD subtype.^41^

While cortical thinning has shown to be a significant characteristic of advancing PD severity, progression, and dementia‐risk stratification,^42^ cortical volume disparities between PD motor subtypes are less clear. While many studies investigating cortical volumes show no difference between PD subtypes,^40^, ^41^, ^43^, ^44^, ^45^ these results could be due to dissimilarities in disease duration, amyloid deposition, and acetylcholine denervation, all of which differentially affect neuronal degeneration.^46^, ^47^ Furthermore, several of the studies that did not find differences between PD subtypes used 1.5 T MRI.^40^, ^43^, ^45^ Subcortical volumes that require higher resolution MRI to image may prove to be more efficacious in volumetrically distinguishing TD from PIGD patients. Nevertheless, alterations in cortical thickness in PD may still be due to divergent etiologies as results show more cortical changes in PIGD when differences were found.^38^

#### *White Matter*

White matter (WM) provides connections between cortical and subcortical GM regions. WM alterations are thought to interfere significantly with postural control due to greater degeneration of complex bilaterally distributed visual, somatosensory, and vestibular systems shown via higher WM signal hyperintensity burden in PIGD compared to TD.^48^ Age‐related white matter changes (ARWMC) have been shown to be lower in TD patients compared to PIGD, and a follow up 2 and 4 years later showed that total ARWMC scores remained lower in TD compared to PIGD patients.^49^ Similarly, studies show that PIGD have reduced white matter integrity compared to non‐PIGD, and that non‐PIGD patients have lower white matter hyperintensity scores (WMHs) when compared to PIGD.^50^, ^51^, ^52^, ^53^ Conversely, a different study showed that the mean number of voxels with WMHs did not differ between TD and PIGD, even when patients with low and high burdens of WMHs were compared.^54^

When TD patients were compared to PIGD, PIGD patients were associated with additional degradations of white matter such as higher leukoaraiosis grade.^53^, ^55^ More recently, when compared to non‐PIGD and HC, PIGD patients showed a significantly higher white matter lesion (WML) load.^56^ In reference 27, researchers also showed PIGD to have higher WML volume compared to TD while reference 57 showed PIGD exhibited more WM degradation relative to TD.

Such white matter alterations are important within PD patients, as a single unit increase in ARWMC score at baseline was associated with a 2.7 times increased likelihood of developing PIGD during a 4‐year observation, showing WM changes may help in defining progression into a specific PD motor subtype at an early stage.^49^ Additionally, significant clinical correlations between WMHs and ratings of posture, as well as borderline correlations of freezing with WM changes further support the view that nTD have worse WM integrity in corticocortical tracts.^48^, ^53^

#### *Iron‐Sensitive Sequences*

Regional iron depositions in deep brain nuclei can be evaluated using phase shifts (radians) derived from filtered MR phase images. Parkinson's symptoms have previously shown to result as a consequence of dopaminergic neurodegeneration, as higher levels of iron measured using substantia nigra (SN) radians have shown to be positively correlated with UPDRS‐III scores as well as bradykinesia‐rigidity subscores, but not with tremor subscores^58^ although TD patients have shown to have higher bilateral dentate nucleus (DN) magnetic susceptibility values compared to AR patients.^2^

Nigral bilateral average phase values and serum ceruloplasmin levels have shown to correlate significantly with each other in both TD and AR.^59^ One study showed PIGD to have lower susceptibility weighted imaging (SWI) intensity values (containing both magnitude and phase information) in all regions compared to non‐PIGD, particularly in the globus pallidus and with a similar trend in other basal ganglia nuclei.^60^ Another study applied NM‐MRI and found that the PIGD subtype had more severe signal attenuation in the medial part of the SNc compared to TD, and that the SNc ipsilateral to the most clinically affected side was the strongest in discriminating the two PD subtypes.^61^ These findings suggest that iron load is involved in the development of bradykinesia and rigidity symptoms such that susceptibility values and NM‐MRI relating to relative iron concentrations could be used to differentiate PD motor subtypes.

#### *Structural Imaging Limitations*

The major limitations of structural imaging study designs to investigate PD involve inconsistent subtype classification and patient selection bias.^25^, ^38^, ^41^, ^53^ Moreover, many lack longitudinal study strategies^33^, ^39^, ^45^, ^48^, ^49^, ^53^, ^54^ and have relatively small sample sizes as summarized in Tables 1 and 2.^33^, ^39^, ^40^, ^45^, ^55^ As MR technology (e.g., ultra‐high field) and imaging techniques advance (e.g., quantitative susceptibility mapping (QSM) that quantifies the magnetic susceptibility value of brain tissue and provides contrast between iron‐rich gray matter nuclei and surrounding tissues), MRI could be used to examine more subtle and subcortical structural changes that occur in PD that cannot be detected with low‐field strengths and current approaches.^2^, ^58^

**TABLE 2:** *Differences with diffusion MRI (dMRI) between PD motor subtypes*

Study	MRI Strength	PD	TD	nTD	PIGD	AR	Mixed	HC	Findings
**Fractional anisotropy**									
(Gu et al., 2014)^66^	3 T GE	12			12				PIGD had reduced FA in bilateral SLF, bilateral anterior corona radiate, and the left genu of the corpus callosum compared to nPIGD. PIGD had increased RD in the left SLF. No significant differences were seen in AD between subtypes.
(Lenfeldt et al., 2016)^65^	1.5 T Philips		40		64		18		TD had higher FA in the EEC and the anterior PFC compared to PIGD.
(Wen et al., 2018)^57^	3 T Siemens		52		13			61	TD showed increased FA, RD, and AD in multiple projection, association, and commissural tracts compared to PIGD.
**Mean diffusivity**									
(Lenfeldt et al., 2013)^67^	1.5 T Philips	36							TD had higher MD in thalamus compared to PIGD.
(Luo et al., 2017)^68^	3 T GE		30	30				26	TD had increased MD and axial diffusivity (AD) along multiple white matter tracts mainly in the cerebello‐thalamo‐cortical (CTC) pathway.
(Nagae et al., 2016)^64^	3 T GE		12		9			20	Compared to TD, PIGD had increased MD in the left and right SN and bilateral GP that correlated with disease stage and motor severity.
(Surova et al., 2016)^70^	3 T Siemens		50		47			44	PIGD had increased MD in the putamen compared to TD.
(Tessa et al., 2008)^45^	1.5 T Siemens		13			11	3	16	TD did not differ in GMV, WMV, or histogram‐derived MD metrics compared to AR.
(Vervoort et al., 2016)^69^	3 T Philips		16		33			18	No differences were shown in FA or MD at the whole‐brain level between patient groups. TD had increased MD in tracts connecting the right M1 and right inferior parietal lobule compared to PIGD. No volumetric differences in caudate, putamen and pallidum between TD and PIGD were seen. No shape alterations in right caudate and the bilateral putamen and pallidum between TD and PIGD were detected.
**Other diffusion metrics**									
(Barbagallo et al., 2017)^62^	3 T GE		35	28				30	TD had higher tract integrity in the globus pallidus–substantia nigra, putamen–precentral cortex, and caudate nucleus–supplementary motor area tracts compared to nTD; TD had higher connectivity values in the thalamus–precentral cortex tract compared to nTD; nTD had structural connectivity alterations of cortico–basal ganglia pathways while TD did not.
(Tan et al., 2019)^51^	3 T Siemens		21		19			20	PIGD had reduced white matter integrity in the superior longitudinal fasciculus, inferior longitudinal fasciculus, inferior fronto‐occipital fasciculus, anterior thalamic radiation, genu of the corpus callosum, and body of the corpus callosum compared to TD.

AD, axial diffusivity; AR, akinetic‐rigid; ECC, external capsule; FA, fractional anisotropy; GE, General Electric; GMV, gray matter volumes; GP, globus pallidus; HC, healthy controls; M1, primary motor cortex; MD, mean diffusivity; Mixed, mixed‐type/intermediate/indeterminate subtype; nTD, non‐tremor‐dominant; PD, Parkinson's disease; PFC, prefrontal cortex; PIGD, postural instability and gait difficulty; RD, radial diffusivity; SLF, superior longitudinal fasciculus; SN, substantia nigra; T, Tesla; TD, tremor‐dominant; WMV, white matter volumes.

### Diffusion Imaging

It has been suggested that microstructural integrity degradations of the BG visible via MR diffusion data play a fundamental role in the underlying neural correlates of TD‐PD symptomologies.^8^ Correspondingly, connectivity indices derived from diffusion images have shown lower structural connectivity in nTD in key neuronal motor areas such as the globus pallidus–substantia nigra tract, globus pallidus–thalamus tract, putamen–precentral cortex tract, thalamus–precentral cortex tract, and the caudate nucleus–supplementary motor area tract compared to TD.^62^

#### *Fractional Anisotropy*

Fractional anisotropy (FA), or the extent that the diffusion of water molecules is restricted or unrestricted in specific directions, is used to denote the *integrity* of white matter within the brain by providing information about myelination, fiber organization, and the number of axons in a single measure.^63^ While an increase in FA could indicate increased myelin, increased axonal density/caliber, or decreased fiber mixture^63^ in general decreased FA along with increases in mean diffusivity (MD) in the SN have pointed towards an ability to distinguish PD patients from healthy controls.^64^

TD patients have shown to have increased FA compared to PIGD patients in multiple projection, association, and commissural tracts, while motor severity was correlated with FA within the corpus callosum of TD patients and even stronger in multiple association tracts within PIGD patients.^57^, ^64^ In ,^64^ PIGD displayed lower FA in the left substantia nigra compared to TD. These studies are in line with others that show TD patients have increased FA compared to PIGD in the external capsule (ECC), anterior PFC, and lateral to the horn of the anterior ventricle^65^ and that PIGD patients have significantly decreased FA in the bilateral superior longitudinal fasciculi (SLF), bilateral anterior corona radiate, and in the left genu (front) of the corpus callosum when compared to non‐PIGD.^66^ These diffusion studies exemplify that the decreased FA found in PIGD are in line with models demonstrating PIGD to have more motor impairments and worse prognosis due to microstructural white matter abnormalities in the cortico‐basal ganglia‐thalamocortical tract.

#### *Mean Diffusivity*

Alongside FA, mean diffusivity (MD) is a diffusion measurement that denotes the average diffusion within a voxel and is used to measure the mobility of water molecules. TD patients have shown increased MD in the thalamus and middle and superior cerebellar peduncle when compared to PIGD^67^, ^68^ as well as increased MD in the tracts connecting the right inferior parietal lobule (IPL) with the right premotor cortex and primary motor cortex^67^, ^69^ and in major white matter tracts including the fornix, longitudinal fasciculi, and corpus callosum.^68^ While one article showed that TD did not differ in histogram‐derived MD metrics compared to AR^45^ and another showed no significant group difference in MD between PIGD and TD,^57^ one showed TD patients had a 7% decrease in MD within the putamen compared to PIGD patients.^70^

These results suggest that while diffusion data shows TD to have deficits in connecting fibers in motor cortical areas, PIGD patients show impaired WM tracts involved in both cognitive and motor control which could partially account for the more severe postural and gait impairments^29^, ^64^ as well as PIGD related incidences of freezing of gait (FOG)^71^ and non‐motor PD symptoms like depression.^72^ Because diffusion parameters can correlate with worse motor and cognitive function in PD^69^ and TD patients seem to have increases in MD compared to the PIGD in certain areas, such WM alterations may underlie the greater impact on motor and non‐motor function seen in PIGD.^71^, ^72^

#### *Diffusion Imaging Limitations*

Since diffusion parameters are sensitive to various microscopic alterations in the brain such as crossing‐fiber mixture, demyelination, and axonal density/caliber, the degree to which the variability in diffusion measurements indicate alternations in PD must be interpreted with caution. Additional limitations across diffusion studies include differences in MRI field strength, sequences used, age of the cohorts, time of disease onset, and sample sizes.^8^, ^65^, ^69^, ^70^ Longitudinal studies are additionally needed to understand the progression of diffusion alterations in PD on white matter microstructure.^57^, ^70^, ^73^, ^74^ Overall, high‐quality diffusion stands as a useful method in differentiating structural aberrations between PD subtypes and serves as an important complement in histological studies that investigate fiber organization and the microstructure of circuitopathies.

### Functional Imaging

#### *Functional Imaging Techniques*

A pivotal brain‐imaging technique is functional magnetic resonance imaging (fMRI) which indirectly measures brain activity via changes associated with cerebral blood flow called a blood‐oxygen level dependent (BOLD) response (Table 3). Outcome measurements of fMRI include functional connectivity (FC) where temporal synchronizations of activity between ROIs reflect communication and correlation during a task, and resting state fMRI (rs‐fMRI) used to calculate interactions between regions while the brain is in a resting state. Furthermore, arterial spin labelling (ASL) is a technique that measures cerebral blood perfusion and allows for the measurement of cerebral blood flow (CBF). Lastly, magnetic resonance spectroscopy (MRS), a non‐invasive technique that quantifies in vivo patterns of neurometabolic alterations, analyzes specific molecules and evaluates metabolites and products of metabolism.^75^

**TABLE 3:** *Differences with functional MRI (fMRI) between PD motor subtypes*

Study	Field Strength	TD	nTD	PIGD	AR	Mixed	HC	Findings
**Functional activity and connectivity**								
(Chen et al., 2015)^76^	3 T Siemens	12		19			22	TD had higher ALFF values in the lobule VIII of the right cerebellum and the bilateral putamen and lower ALFF values in the bilateral temporal gyrus and the left superior parietal lobule compared with PIGD.
(DiMarzio et al., 2020)^83^	1.5 T, 3 T GE	11		7	5			AR showed more activation in primary motor cortex (M1) compared to TD.
(Helmich et al., 2011)^34^	3 T Siemens	21	23				36	TD had enhanced GPi‐MC and putamen‐MC coupling compared to nTD, mainly in the MAH.
(Hou et al., 2018)^88^	3 T GE	21		19			35	TD had higher FC between the BG and calcarine region (occipital lobe) compared to PIGD.
(Hu et al., 2017)^15^	3 T Siemens	25			25		26	TD showed significantly decreased global FCD in the left inferior frontal gyrus, right middle frontal gyrus and right superior frontal gyrus, and increased global FCD in the cerebellum anterior lobe compared to AR.
(Hu et al., 2019)^89^	3 T Siemens	16		23			28	TD showed increase ReHo values in the right para‐hippocampal gyrus compared to PIGD.
(Jiang et al., 2016)^78^	3 T Siemens	15		13			17	TD exhibited lower ReHo in the left precuneus, left S1, left cuneus and left superior occipital gyrus and higher ReHo values in the right lingual gyrus, right parahippocampal gyrus, and cerebellum
(Karunanayaka et al., 2016)^39^	3 T Siemens	15			17		24	AR had lower activation in the left IPC and left PCC compared to TD and controls.
(Lewis et al., 2011)^79^	3 T Siemens	9			8		14	TD had decreased activation in all STC and CTC circuits expect for the ipsilateral STC compared to AR. TD had decreased activity in the lentiform nucleus of the BG compared to AR. TD had higher activity in cerebellular and thalamic regions compared to AR.
(Liu et al., 2013)^80^	3 T GE	10			8		18	TD had decreased positive correlations of the left and right DN with the bilateral CPL compared to AR.
(Ma et al., 2017)^81^	3 T Siemens	12		19		14	22	TD had higher FCS values in the cerebellum, mainly in the left hemisphere and tonsils compared to PIGD.
(Mohl et al., 2017)^82^	3 T GE	14		12			21	PIGD ON had increased activation in the right superior temporal gyrus compared to TD ON.
(Prodoehl et al., 2013)^44^	3 T GE	10	10				20	TD had higher activation in the bilateral DLPFC, contralateral pre‐SMA, ipsilateral IPL, ipsilateral precuneus, contralateral lingual gyrus, contralateral caudate, contralateral (GPi, GPe), and ipsilateral thalamus compared to nTD. nTD did not show increased activity in any area compared to TD.
(Shen et al., 2020)^87^	3 T Siemens	16		23			28	TD showed increased FC between the left putamen and right cerebellum lobule VI and cerebellum crus I compared to PIGD.
(Vervoort et al., 2016)^71^	3 T Philips	16		33			18	PIGD had decreased FC in the striatum (eg, between the left caudate and bilateral ventral and dorsal putamen) and lower FC between the left caudate and right pallidum and between the left caudate and left PMC and right STL. No increases of FC were found in PIGD compared to TD.
(Zeng et al., 2019)^86^	3 T GE	43		36			31	TD showed to have greater connectivity between the bilateral Vim and the bilateral cerebellum compared to PIGD.
(Zhang et al., 2014)^17^	1.5 T Siemens	15			10		20	The ability to distinguish TD from nTD using network nodal efficiency (ie, local and global efficiencies) was heavily influenced by the cerebellum.
**Resting‐state fMRI**								
(Baudrexel et al., 2011)^85^	3 T Siemens	16	15				44	No direct statistical difference in STN FC or left STN‐M1 hand area FC between TD and nTD.
(Hu et al., 2015)^90^	3 T Siemens	21			29		26	TD had decreased VHMC in the posterior lobe of the cerebellum compared to AR.
(Jiang et al., 2016)^78^	3 T Siemens	12		13			17	TD had lower ReHo values in the left precuneus, left S1, left cuneus and left superior occipital gyrus, and higher ReHo values in the right lingual gyrus, right parahippocampal gyrus, and cerebellar areas compared to PIGD.
(Wang et al., 2016)^84^	3 T Siemens	12		19			22	PIGD had greater STN FC with the left middle occipital lobe, left superior parietal lobe, and right middle frontal lobe. TD had greater FC between the bilateral STN and left cerebellar anterior lobe and right middle cingulate gyrus.
**Cerebral blood flow**								
(Al‐Bachari et al., 2017)^27^	3 T Philips	21		24		6		TD did not differ in whole brain CBF compared to PIGD. TD had more hypoperfusion in temporo–parieto–frontal networks compared to PIGD, while PIGD showed hypoperfusion in a predominantly posterior pattern as well as hyperperfusion in the BG
**Other functional metrics**								
(Barbagallo et al., 2017)^93^	3 T GE	14	12 (rET)				10	TD showed reductions of NAA/Cr and Cho/Cr in the thalami compared to rET and HC. The combination of thalamic NAA/Cr and Cho/Cr ratios showed a 100% accuracy in distinguishing TD from rET and HC. No differences were seen regarding Glu/Cr ratios.

ALFF, amplitude of low‐frequency fluctuation; AR, akinetic‐rigid; BG, basal ganglia; CBF, cerebral blood flow; Cho, glycerophosphocholine with phosphocholine; CPL, cerebellar posterior lobe; Cr, creatine with phosphocreatine; CTC, cerebello‐thalamo‐cortical circuit; DLPFC, dorsolateral prefrontal cortex; DN, dentate nucleus; FC, functional connectivity; FCD, Functional connectivity density; FCS, functional connectivity strength; GE, General Electric; Glu, glutamate with glutamine; GPe, external globus pallidus; GPi, internal globus pallidus; HC, healthy controls; IPC, inferior parietal cortex; IPL, inferior parietal lobule; M1, primary motor cortex; MAH, most affected hemisphere; MC, motor cortex; Mixed, mixed‐type/intermediate/indeterminate subtype; NAA, N‐acetyl‐aspartate with N‐acetyl‐aspartyl‐glutamate; nTD, non‐tremor‐dominant; ON, on Levodopa medication; PCC, posterior cingulate cortex; PIGD, postural instability and gait difficulty; PMC, premotor cortex; rET, essential tremor with resting tremor; ReHo, regional homogeneity; S1, right postcentral gyrus; SMA, supplementary motor cortex; STC, striato‐thalamo‐cortical; STL, superior temporal lobe; STN, subthalamic nucleus; T, Tesla; TD, tremor‐dominant; VHMC, voxel‐mirrored homotopic connectivity.

### **fMRI**

#### *Functional Activity and Connectivity*

Many studies have shown task‐based functional alterations between TD patients and nTD within the cerebellum, the putamen, the temporal cortex, and the parietal cortex.^34^, ^39^, ^44^, ^74^, ^76^, ^77^, ^78^, ^79^, ^80^, ^81^, ^82^ Other areas have also shown to have functional differences between motor subtypes including TD having enhanced GPi–motor cortex (MC) and putamen–MC coupling compared to nTD, mainly in the most‐affected hemispheres (MAH),^34^ PIGD having lower FC (i.e., more disrupted hubs) in the cerebellum, mainly in the left hemisphere and tonsils compared to TD,^81^ and nTD showing reduced BOLD activity in the PFC and GP compared to TD.^44^

Compared to TD, nTD have also shown reduced activation in bilateral dorsolateral PFC, contralateral pre‐supplementary motor area, ipsilateral IPL, ipsilateral precuneus, contralateral caudate, contralateral GPi and GPe, and the ipsilateral thalamus during a gripping task, while no areas in nTD showed increased activity compared to TD, showing that even in the earliest stages of PD nTD show greater deficits in frontal cortical areas compared to TD.^44^ Furthermore, when compared to TD, AR have shown to have increased activation during sequential finger tapping tasks in cortical and subcortical ROI related to PD such as the lentiform nucleus of the basal ganglia, as AR showed increased activity in contralateral CTC circuits while TD showed significant differences in the contralateral striato‐thalamo‐cortical circuit (STC) and CTC pathways including the cerebellar vermis, contralateral cerebellar hemisphere, and ipsilateral thalamus.^79^ Likewise, a recent study with patient's deep brain stimulation (DBS) cycling ON and OFF showed AR have increased activation in the supplementary motor area (SMA) and primary motor cortex (M1) compared to TD.^83^

#### *Resting‐State fMRI*

Using rs‐fMRI, PIGD have shown less subthalamic nucleus (STN) FC within the left anterior and posterior lobes of the cerebellum, less FC between the bilateral STN and left cerebellar anterior lobe and right middle cingulate gyrus, but greater FC between the STN and the left middle occipital lobe, left superior parietal lobe, and right middle frontal lobe compared to TD.^84^ Conversely,^85^ found no significant differences in STN FC between TD and nTD.

In reference 17, the ability to functionally distinguish TD and nTD was influenced by the cerebellum, while in 15 TD showed increased global functional connectivity density (FCD) in the cerebellum anterior lobe relative to AR. In 86, TD showed to have greater connectivity between the bilateral ventral intermediate nucleus (Vim) and the bilateral cerebellum compared to PIGD while reference 87 showed TD to have increased FC between the left putamen and right cerebellum lobule VI and cerebellum crus I compared to PIGD. TD has also shown to have higher FC between the BG and calcarine region (occipital lobe) compared to PIGD.^88^ In a later study, PD patients with FOG showed decreased FC between the left caudate and the right superior temporal lobe (STL) and left cerebellum, between the right caudate and bilateral dorsal putamen, left GP, and bilateral STL, and increased FC between the right precuneus and the left dorsal putamen compared to those without FOG.^74^

Using amplitude of low frequency fluctuations (ALFF) which detect the regional intensity of spontaneous fluctuations in BOLD signals,^76^ found TD to have increased ALFF in the putamen and the posterior lobes of cerebellum compared to PIGD, and decreased ALFF in the temporal gyri and left superior parietal lobule. In another study using low frequency rs‐fMRI, TD had decreased correlation of the left and right DN with the bilateral posterior lobe of cerebellum compared to AR.^80^

TD have also shown more regional homogeneity (ReHo) alterations, a resting‐state analysis that examines synchronizations of temporal changes in BOLD signal, in the cerebellum, right para‐hippocampal gyrus, and CTC loops while PIGD showed increased ReHo values in areas involved in the STC loop including in the frontal, parietal, occipital, temporal, and limbic lobes, basal ganglia, and thalamus.^78^, ^89^ Lastly, compared to AR, TD have shown lower voxel‐mirrored homotopic connectivity (VMHC) values, which denote synchrony in patterns of spontaneous rs‐fMRI activity, in the posterior lobe of the cerebellum.^90^

These results show that fMRI and rs‐fMRI are valuable imaging techniques to better understand functional differences in PD subtypes and further underline the importance of cerebellular and basal nuclei activity as well as the STC and CTC tracts in functional PD imaging, with^86^ recently denoting the cerebellar‐receiving nucleus of the thalamus, the Vim, as a “key nodal point” in both PD subtypes. It seems that the dysfunction of the STC seen in bradykinesia and rigidity and the primary dysfunction of the CTC in TD are the key functional deficits between PD subtypes.^79^ These results are in line with structural findings and support network models of PD subtypes.

#### *Cerebral Blood Flow*

Cerebral blood flow (CBF) is the movement of blood in arteries and veins within the brain and is an important marker of PD as it maintains proper brain function by supplying the brain with oxygen and energy substrates that remove waste products of metabolism.^91^ A recent study using ASL showed TD to have more hypoperfusion in the temporo–parieto–frontal network while PIGD showed hypoperfusion in a predominantly posterior pattern as well as hyperperfusion in the BG, although these differences were removed when levodopa medication, and disease severity and duration were controlled for.^27^ Comparatively, in a recent structural study PIGD were associated with an increased prevalence of thalamic and WM cerebral microbleeds (ie, small chronic brain hemorrhages caused by abnormalities of small brain vessels) when compared to TD and AR.^92^ These results suggest that CBF and other cerebral blood parameters could be valuable imaging techniques to differentiate between PD subtypes.

#### *Other Functional Metrics*

One study that used proton MR spectroscopy (^1^H‐MRS) reported TD patients had reduced N‐acetyl‐aspartate (NAA)/creatine (Cr) and glycerophosphocholine (Cho)/Cr ratios in the ipsi‐ and contralateral thalami compared to patients with essential and resting tremor (rET) and to healthy controls.^93^ Although rET is not a PD subtype, NAA/Cr and Cho/Cr ratios were 100% accurate at differentiating TD from rET and controls^93^ showing that TD PD can be differentiated from those with postural and kinetic tremors using MRS which could help with diagnostics during the early stages of these diseases. Therefore, MRS might have the potential to accurately classify PD subtypes.

### Limitations of fMRI

Dissimilar and low resolution MR, variations in cohort disease stage, and small sample sizes limit the generalization of functional imaging findings across PD subtypes. Furthermore, artifacts in the BOLD signal from head motion originating from tremor symptoms are significant limiting factors across PD fMRI studies. Nevertheless, functional MRI, and especially rs‐fMRI, hold legitimate potential for better characterizations of PD subtypes and have translational applications for clinical and psychotherapeutic PD domains.

## Molecular Imaging

### Molecular Imaging Techniques

Little is known about the differences in metabolism and disrupted molecular processing within identical brain regions between PD subtypes.^34^, ^94^ Functional nuclear medicine tomographic imaging techniques are used to investigate such alterations on a molecular level such as positron emission tomography (PET) that uses positron emitting radioisotopes (Table 4), and single photon emission computed tomography (SPECT) that can differentiate between isotopes with different energy levels (Table 5).

**TABLE 4:** *Differences with positron emission tomography (PET) imaging between PD motor subtypes*

Study	Company	TD	nTD	PIGD	nPIGD	AR	Mixed	HC	Radiotracer	Findings
**Dopaminergic**										
(Eggers et al., 2014)^94^	Siemens	32				32			[^18^F]F‐DOPA	AR had reduced dopaminergic uptake in the caudate and anterior putamen compared to TD patients.
(Lee et al., 2019)^100^	X	221		75			29		I‐^123^ FP‐CIT	Specific binding ratios of the caudate and putamen were significantly lower in the PIGD group than the TD group.
(Song et al., 2014)^95^	GE	21	22					18	^18^F‐FP‐CIT	TD did not differ in DAT uptake in the striatum compared to nTD at the same early stage of disease
**Non‐dopaminergic**										
(Ahrweiller et al., 2019)^102^	GEMS			10	46				^18^F‐FDG	PIGD showed increased metabolism in the dorsal midbrain/pons and right motor cerebellum compared to non‐PIGD.
(Antonini et al., 1998)^101^	GE	8	8					10	^18^F‐FDG	PDRP expression did not differ between TD and nTD.
(Eggers et al., 2014)^94^	Siemens	32				32			^18^F‐FDG	Global FDG‐metabolism did not differ between AR and TD. AR showed lower glucose metabolism in the striatal areas compared to TD.
(Loane et al., 2013)^104^	Siemens	12				12		12	^11^C‐DASB	TD had lower serotonin transporter uptake in the caudate and putamen compared to AR. TD trended to have lower binding values in the raphe nuclei compared to AR.
(Zhang et al., 2016)^103^	Siemens	15		15				17	^18^F‐FDG	Compared to TD patients, PIGD had more metabolic decreases in caudate and inferior parietal lobule (IPL, BA 40).

[^18^F]F‐DOPA, ^18^F‐radiolabeled nonproteinogenic amino acid 3,4‐dihydroxy‐6‐[F]fluoro‐l‐phenylalanine; ^18^F‐FDG, 2‐deoxy‐2‐[F]fluoro‐D‐glucose; ^18^F‐FP‐CIT, Fluorinated N‐3‐fluoropropyl‐2β‐carbomethoxy‐3β‐(4‐iodophenyl) nortropane; AR, akinetic‐rigid; BA, Brodmann area; DAT, dopamine transporter; GE, General Electric; GEMS, GE Signa scanner; HC, healthy controls; I‐^123^ FP‐CIT, Ioflupane (FPCIT); [I‐12] N‐ω‐fluoropropyl‐ 2β‐carbomethoxy‐ 3β‐(4‐iodophenyl) nortropane; IPL, inferior parietal lobule; Mixed, mixed‐type/intermediate/indeterminate subtype; nPIGD, non postural instability and gait difficulty; nTD, non‐tremor‐dominant; PDRP, Parkinson's disease ‐related pattern *“characterized by relative hyper‐metabolism of the lentiform nucleus and the thalamus associated with metabolic decreases in the primary and association motor cortices”* (Antonini et al.)^101^; PIGD, postural instability and gait difficulty; TD, tremor‐dominant; X, Images were taken from the Parkinson's Progression Markers Initiative (PPMI) database and presumably come from various company's machines.

**TABLE 5:** *Differences with single‐photon emission computerized tomography (SPECT) imaging between PD motor subtypes*

Study	Company	TD	nTD	PIGD	AR	Mixed	HC	Radiotracer	Findings
**Dopaminergic**									
(Barbagallo et al., 2017)^93^	GE	14	12 (rET)				10	[^123^I]FP‐CIT	DAT was abnormal in TD but normal in rET patients.
(Barbagallo et al., 2017)^62^	GE	35	28				30	[^123^I]FP‐CIT	nTD had lower uptakes values in the less affected side caudate nucleus compared to TD
(Eggers et al., 2011)^97^	GE	23			23			[^123^I]FP‐CIT	TD and AR matched for age, disease duration, and disease severity did not differ in dopaminergic uptake in the caudate and the putamen.
(Eggers et al., 2012)^98^	GE	14			13			[^123^I]FP‐CIT	AR showed reduced dopaminergic uptake in the contralateral caudate and the ipsi‐ and contralateral putamen over time, while TD did not show differences in the same period.
(Helmich et al., 2011)^34^	Siemens	21	23				36	[^123^I]FP‐CIT	TD had enhanced GPi‐MC and putamen‐MC coupling compared to nTD in the most‐affected hemisphere. TD had lower pallidal DAT binding in the most‐affected hemisphere and higher pallidal DAT binding in the least‐affected hemisphere compared to nTD. TD trended to have higher overall DAT binding compared to nTD.
(Isaias et al., 2007)^110^	GE	10			10			[^123^I]FP‐CIT	TD had lower ipsilateral striatum and caudate nucleus uptake compared to AR. TD did not differ in putamen mean uptake values, asymmetry index, or putamen caudate index values compared to AR.
(Kaasinen et al., 2014)^111^	GE	157	74				230	[^123^I]FP‐CIT	TD had higher mean caudate nucleus uptake and left caudate nucleus uptake compared to nTD.
(Mo et al., 2010)^109^	GE	39		75		14	48	[^123^I]FP‐CIT	TD had higher presynaptic putamen uptake ratios compared to PIGD even when controlling for disease severity. TD also had higher presynaptic caudate uptake ratios compared to PIGD but not after disease severity was controlled for.
(Moccia et al., 2014)^105^	GE	27			24			[^123^I]FP‐CIT	TD had higher DAT availability in the affected and unaffected putamen but no differences in the caudate.
(Qamhawi et al., 2015)^99^	GE	37			106	202	185	[^123^I]FP‐CIT	TD had less severe striatal dopaminergic deficits compared to nTD.
(Ramani et al., 2017)^112^	GE	7			17	18		[^123^I]FP‐CIT	TD did not differ in baseline uptake ratios compared to AR and Mixed.
(Rossi et al., 2010)^106^	GE	24			38			[^123^I]FP‐CIT	TD had higher average uptake compared to AR. TD had higher FP‐CIT uptake in the putamen contralateral to the most clinically affected side compared to AR, but no differences were seen in bilateral caudate and ipsilateral putaminal uptakes, or in asymmetry indices and caudate or putamen ratios.
(Schillaci et al., 2011)^107^	GE	20			20	7		[^123^I]FP‐CIT	TD had higher uptake in the caudate contralateral to the side affected compared to AR and Mixed, while no differences were seen between AR and Mixed. TD had higher uptake in the contralateral putamen compared to AR, but not compared to Mixed, nor when AR was compared to Mixed. TD had higher uptake in the ipsilateral caudate compared to AR and Mixed, while no difference were seen when AR was compared to Mixed. TD had higher uptake in the ipsilateral putamen compared to AR. No differences were seen between TD and Mixed, or between AR and Mixed in ipsilateral putamen uptake.
(Spiegel et al., 2007)^108^	Siemens	22			26	19		[^123^I]FP‐CIT	TD had higher uptake in the ipsilateral and contralateral putamen compared to AR and Mixed. AR and Mixed did not differ in their binding ratios in the contra‐ or ipsilateral putamen. TD had higher uptake in the ipsilateral and contralateral caudate nucleus compared to AR and Mixed. AR and Mixed did not differ in their binding ratios in the contra‐ or ipsilateral caudate nucleus or contra‐ or ipsilateral putamen.
**Non‐dopaminergic**									
(Caretti et al., 2008)^113^	Strichmann	8,6	12,11				13	[^123^I]β‐CIT	TD had 19% lower SNS thalamic binding ratios compared to nTD at baseline. TD did not differ in whole striatum, putamen, and caudate nucleus average SNS binding ratios compared to nTD at baseline nor at follow up. There were no differences in thalamic binding ratios between nTD and TD at follow‐up.
(Mito et al., 2006)^114^	Toshiba	10		19			17	Iofetamine (^123^I)	TD did not differ in patterns of regional cerebral blood flow (rCBF) when directly compared to PIGD.
(Qamhawi et al., 2015)^99^	GE	37			106	202	185	[^123^I]FP‐CIT	TD had lower raphe serotonin transporter availability compared to nTD.

Note: In Caretti et al., (2008) multiple numbers reflect baseline cohort and 17 month follow up cohorts.

[^123^I]FP‐CIT, N‐(3‐Fluoropropyl)‐2β‐carbomethoxy‐3β‐(4‐[I]iodophenyl)nortropane; [^123^I]β‐CIT, Iodine‐123‐β‐carbomethoxy‐3 β‐(4‐iodophenyltropane); AR, akinetic‐rigid; DAT, dopamine transporter; GE, General Electric; GPi, internal globus pallidus; HC, healthy controls; Iofetamine (^123^I), N‐isopropyl‐(123I)‐p‐iodoamphetamine; MC, motor cortex; Mixed, mixed‐type/intermediate/indeterminate subtype; nTD, non‐tremor‐dominant; PIGD, postural instability and gait difficulty; rCBF, regional cerebral blood flow; rET, resting essential tremor; SNS, specific to non‐specific; TD, tremor‐dominant.

### PET Imaging

#### *Dopaminergic PET Imaging*

The clinical expression of PD can be partially explained by dopamine transporter (DAT) loss localized in presynaptic nigrostriatal nerve terminals. Most PET studies used ^18^F‐FP‐CIT (N‐3–fluoropropyl‐2‐b‐carboxymethoxy‐3‐b‐(4‐iodophenyl) nortropane) as a radioligand for dopamine receptors and re‐uptake sites due to its fast kinetics, relatively long half‐life, and low radiation exposure as compared to other radioligands. The main ROI is the striatum with subregions defined as the caudate and putamen (both split into anterior and posterior parts). Compared with a HC group, PD patients overall show a reduced striatal ^18^F‐FP‐CIT binding in the caudate and putamen.^95^, ^96^

Although reference 97 showed no significant differences in dopaminergic uptake between TD and AR, two later studies of theirs showed TD had increased dopaminergic uptake in the caudate and anterior putamen compared to AR patients^94^, ^98^ and a separate study showed TD to have less severe striatal dopaminergic defects compared to AR.^99^ Further comparisons between TD and PIGD show increased dopamine uptake in TD in the caudate, putamen, and IPL (Brodmann area [BA] 40),^100^ and although one study showed no differences in FP‐CIT uptake in the striatum between the TD and nTD^95^ another reported TD to have enhanced GPi–MC and putamen–MC coupling compared to nTD in the MAH.^34^

#### *Non‐Dopaminergic* *PET Imaging*

Early work using ^18^F‐fluorodeoxyglucose (FDG) PET imaging showed that PD patients had increased metabolic activity in the motor association cortices, pons, and thalamus.^101^ In another study of PD patients who underwent subthalamic nucleus deep brain stimulation and subsequent PET scans using FDG, PIGD showed increased metabolism in the dorsal midbrain/pons and right motor cerebellum compared to non‐PIGD.^102^ Separate studies show increased glucose uptake in the ventral striatum in TD compared to AR,^94^ PIGD having metabolic decreases in the caudate and inferior parietal lobule (Brodmann area (BA) 40) compared to TD,^103^ and TD having lower raphe serotonin transporter availability.^99^ Another study investigated the vesicular monoamine transporter type 2 (VMAT2) binding with [^11^C]dihydrotetrabenazine as a tracer and showed a significant covariate effect of VMAT2 when comparing TD with AR.^53^ Furthermore, using ^11^C‐labeled 3‐amino‐4‐[2‐[(di(methyl)amino) methyl]phenyl]sulfanylbenzonitrile (^11^C‐DASB) to investigate serotonin transporter uptake, one study showed lower uptake in the caudate and putamen in the TD compared to the AR, and TD trended to have lower raphe nucleus ^11^C‐DASB values compared to AR.^104^ In addition to these findings, the study also reported reductions of ^11^C‐DASB uptake in the thalamus and in BA 4 and 10 in TD compared to AR with a voxel‐based analysis.

#### *PET Limitations*

Motor impairments in PD cannot be fully explained by PET findings, as complex comorbid deficits and the degeneration of other neuronal systems occur simultaneously.^48^, ^53^ Some studies focus on local glucose metabolism while others look at whole brain analysis, making multimodal imaging techniques necessary to consolidate PD specific degenerations found via PET imaging. As summarized in Table 4, there seems to be an increase in dopamine uptake in the TD group compared to the nTD group.

### SPECT Imaging

#### *Dopaminergic SPECT Imaging*

SPECT studies in PD make use of ^123^I‐FP‐CIT for tracing dopamine uptake in the striatum. Based on available literature it can be noted that TD compared to nTD show higher uptake in the putamen contralateral to the MAH.^62^, ^97^, ^105^, ^106^, ^107^, ^108^ TD compared to nTD show higher uptake on the ipsilateral side,^62^, ^97^, ^107^ and TD show higher uptakes when the means of the right and left uptake ratios of the putamen were compared between groups as well.^99^, ^109^ Differences in the striatum support previous neuropathological models for PD motor subtypes in vivo, where AR have reduced dopaminergic projections to the dorsal putamen and TD have reduced projections in the lateral putamen and caudate nucleus.^97^ Contrary to these findings, several SPECT studies found no difference between motor subtypes in the anterior or posterior putamen.^110^, ^111^, ^112^ Interestingly, there seems to be a differential pattern of progression in the FP‐CIT binding in the ipsi‐ and contralateral putamen, since nTD had decreased binding over time, while TD showed no differences.^98^ One study reported PIGD to have lower striatal presynaptic ratios as PIGD were seen to be more affected by the disease than TD.^109^

While the putamen region is the most examined region in PD SPECT studies, few studies report on other dopaminergic regions. When TD was compared against AR, higher uptake was found in the ipsilateral and contralateral caudate nucleus,^97^, ^107^, ^108^ and in mean caudate uptake,^111^ while two studies found no difference in contralateral or ipsilateral caudate binding ratios.^105^, ^112^ Contrary to the findings of higher ipsilateral FP‐CIT uptake in the caudate nucleus, one study found lower ipsilateral striatum and caudate nucleus uptake in TD compared to AR^110^ and another showed PD subtypes with the same severity of disease show no difference in caudate uptake ratios.^109^

#### *Non‐Dopaminergic SPECT Imaging*

One study that used [^123^I]β‐CIT binding to measure serotonin reported a 19% higher binding ratio in the thalamus in nTD compared to TD but no differences in binding ratios within the striatum, putamen, or caudate nucleus.^113^ When raphe nuclei serotonin transporter availability was investigated using ^123^I‐iodoamphetamine, TD showed significantly lower uptake compared to AR.^99^ Lastly, when the mean brain CBF, deemed regional CBF (rCBF) was examined with SPECT, one study found TD had no significant decreases in rCBF compared to PIGD in any region.^114^

#### *SPECT Limitations*

The asymmetric findings, as summarized in Table 5, are of important note as bilateral and interhemispheric differences in PD are a fundamental aspect of the disease. As these findings are mixed, whereas TD show less FP‐CIT uptake in some neuronal areas and more in others compared to nTD, SPECT should be used in combination with other techniques to distinguish PD subtype etiology.

## Discussion

As variable presentations of motor symptoms suggest divergent pathophysiological, anatomical, and neurochemical mechanisms during the course of PD progression^62^, ^93^, ^114^ neuroimaging is a valuable tool towards identifying neuronal alterations and predicting symptom manifestation. To our knowledge, the current paper is the first to review studies of diverse neuroimaging alterations between TD‐PD patients and those with nTD motor subtypes. Neuroimaging has shown variability between TD and nTD PD patients and persistently supports the notion that the subtype of TD is the more “benign” subtype as TD shows less negative alternations compared to nTD.

Importantly, while nTD have shown symptoms that are more aggressive compared to TD, revealed by earlier and more rapid physical decline,^16^, ^29^ circuitry theories of how PD tremor is generated have only been minimally investigated within different nTD PD subtypes. The literature reviewed here shows that nTD patients have deficits within striato‐thalamo‐cortical (STC) circuitry and other thalamocortical projections related to cognitive and sensorimotor function, while TD patients show greater cerebello‐thalamo‐cortical (CTC) circuitry dysfunction. Comparatively, structural connectivity analysis show nTD have alterations of cortico–basal ganglia pathways while TD do not.^62^ This is in line with the *“dimmer‐switch model*” of PD resting tremor, that suggests pathological activity in the STC from dopaminergic denervation of the GP triggers tremor‐related responses in the CTC via the motor cortex where both circuits converge; the BG acts as a light switch triggering tremors on and off, while the CTC modulates the tremors intensity similar to a light dimmer.^34^ The results further support studies showing depletion of nigrostriatal dopamine and subsequent BG dysfunction alone is insufficient to characterize TD pathology fully^5^, ^115^, ^116^ as other neuronal systems such as the cerebellum play ample roles in the production of tremors.^34^, ^117^ Activity in the BG and cerebellum has shown to be highly associated and structurally connected via the thalamus and pontine nucleus.^117^ When targeted surgically with DBS, the Vim has shown to produce relief of tremor^21^ and activity in the Vim that receives projections from the cerebellum, as well as from the GPi, has shown to synchronize with, mediate, and be directly related to tremor activity.^118^, ^119^, ^120^, ^121^ These finding are consistent with reports that the GP and putamen in TD patients have increased connectivity with the Vim–motor cortex–cerebellum circuit via the motor cortex^34^ and further support results showing that a combination of STC and CTC circuitry might be behind the generation of tremors in PD.^17^

Complementary neuroimaging techniques are required to isolate neural mechanisms underlying PD motor symptomologies that can be used as non‐invasive biomarkers in assessing PD trajectories and responses to treatment. Altogether, there remains an urgent need for more complete consolidation of macro/microstructural, functional, perfusion, chemical, and metabolic data from dissimilar PD cohorts to aid in refining antemortem diagnoses and improve epidemiological and clinical‐therapeutic trial designs.

## Author Roles

(1) Research project: A. Conception, B. Organization, C. Execution; (2) Statistical Analysis: A. Design, B. Execution, C. Review and Critique; (3) Manuscript: A. Writing of the first draft, B. Review and Critique; (4) Literature Search: A. Design, B. Execution.

J.B.: 1A, 1B, 1C, 3A, 3B, 4A, 4B

S.M.: 1A, 1B, 1C, 3A, 3B, 4A, 4B

Y.T.: 1A, 3B

G.H.: 1A, 1B, 3B

A.J.: 1A, 1B, 3B

## Disclosure

**Ethical Compliance Statement:** The authors confirm that the approval of an institutional review board and patient consent was not required for this work. We confirm that we have read the Journal's position on issues involved in ethical publication and affirm that this work is consistent with those guidelines.

**Funding Sources and Conflicts of Interest:** This work was funded by a Stichting de Weijerhorst Research grant to YT and AJ. The authors whose names are listed above report NO affiliations with or involvement in an organization or entity with a financial or non‐financial interest in the subject matter or materials discussed in this manuscript, report no conflicts of interest, and hereby allow this information to be disclosed to learners in prin.

**Financial Disclosures for the Previous 12 Months:** The authors have no disclosures to report.

## Supporting information


**File S1.** PubMed String Search. PubMed string search based on various dictions of Parkinson's disease (PD), neuroimaging techniques (MRI), and PD subtypes (TD, nTD, PIGD, AR) built using Medical Subject Headings (MeSH), additional potential terms, and PubMed search tools.
