# Supplementary material for: Neuroimaging Detectable Differences between Parkinson's Disease Motor Subtypes: A Systematic Review
Source: Mov Disord Clin Pract. 2020 Nov 6;8(2):175–92. doi: 10.1002/mdc3.13107 (PMC7853198; doi:10.1002/mdc3.13107)
Supplement: Supplementary file 1 — File S1. PubMed String Search. PubMed string search based on various dictions of Parkinson's disease (PD), neuroimaging techniques (MRI), and PD subtypes (TD, nTD, PIGD, AR) built using Medical Subject Headings (MeSH), additional potential terms, and PubMed search tools. [file MDC3-8-175-s001.docx]

**PubMed String Search:**

**("**parkinson disease"[MeSH] OR "parkinsonian disorders"[MeSH] OR "lewy body disease"[MeSH] OR ("parkinson disease"[MeSH terms] OR ("parkinson"[all fields] AND "disease"[all fields]) OR "parkinson"[All Fields] OR "parkinson disease"[all fields] OR ("parkinson's"[all fields] AND "disease"[all fields]) OR "parkinson's"[All fields])) AND ("magnetic resonance imaging"[MeSH Terms] OR ("magnetic"[All Fields] AND "resonance"[All Fields] AND "imaging"[All Fields]) OR "magnetic resonance imaging"[All Fields] OR "mri"[All Fields] OR ("fMRI"[All Fields] OR "resting state"[All Fields] OR "rsfMRI"[All Fields] OR "rs-fMRI"[All Fields]) OR ("DWI"[All Fields] OR "DTI"[All Fields] OR "DMRI"[All Fields]) OR ("PET"[All Fields] OR "SPECT"[All Fields])) AND ("motor subtypes"[All Fields] OR ("motor"[All Fields] AND "subtype"[All Fields] OR "subtypes"[All Fields] OR "subgroups"[All Fields] OR "phenotype"[All Fields] OR "phenotypes"[All Fields]) OR ("postural instability and gait disorder"[All Fields] OR ("postural"[All Fields] AND "instability"[All Fields] AND "gait"[All Fields]) OR "PIGD"[All Fields]) OR ("akinetic-rigid"[All Fields] OR "akinetic"[All Fields] OR "rigid"[All Fields] OR "rigidity"[All Fields] OR "akinetic-rigidity"[All Fields] OR hypokinesia-rigidity[All Fields] AND Fields[All Fields] AND ("tremor"[MeSH Terms] OR "tremor"[All Fields]) AND Fields[All Fields] AND (("tremor"[MeSH Terms] OR "tremor"[All Fields]) AND dominant[All Fields]) AND Fields[All Fields] AND tremor-dominant[All Fields] AND Fields[All Fields] AND (non[All Fields] AND ("tremor"[MeSH Terms] OR "tremor"[All Fields]) AND dominant[All Fields]) AND Fields[All Fields] AND non-tremor[All Fields])) AND ("humans"[MeSH Terms] AND English[lang])
